# Cerebro-Spinal Meningitis

**Published:** 1849-06

**Authors:** 


*Cerebro-spinal Meningitis.* By A. Stone, M. D., Auburn, Mass., March 30, 1849.

This disease has prevailed to a frightful extent, for the last four weeks, in the towns of Sutton and Millbury, in this State. The first case was that of a boy 10 years of age, in Sutton. He was suddenly attacked with entire prostration of reason, delirium, intense pain, spasms, great coldness of extremities and whole body, with collapse of the vital energies. After a rather imperfect re-action had taken place, petechiæ, or a mottled state of the skin and surface, made its appearance, which led to the conclusion that the character of the disease was a more aggravated form of typhus, or the spotted fever that ravaged portions of New England in 1810, and subsequent years. But other cases occurring in rapid succession, and with such fatality, led to the suspicion of a more alarming and deadly disease than even that fatal malady.

The prevailing features in this epidemic are strikingly coincident with that which has prevailed so extensively and fatally in France and Ireland, and other portions of Europe. The majority of cases have been among boys, but according to the number, more male and female adults have been its victims than in Europe. One case, a boy, 5 years old, terminated fatally in the short space of ten hours from the time of the first discovered symptoms, one case in thirteen hours; and others in twenty-four hours. This, added to the circumstance that males and females of upwards of 60 years old have been its victims, would make it appear to be of a more mortal character than either of the epidemics of Europe—as the shortest period there, of termination, out of hundreds, was fifteen hours.

With one or two exceptions, every patient has been suddenly prostrated with loss of reason, rigors, extreme coldness of the extremities, and lividness, loss of vital warmth, pulse small and thready, severe pain, convulsions and spasms, especially of the neck, attended with rigidity. The state of delirium and restlessness has been so extreme, in most cases, as to require from four to six attendants to restrain the patient. In those patients that continue for three or four days or more, re-action, more or less perfectly, takes place; the pulse is irregular, small, tremulous and intermitting; the pupils dilate or contract, according to exposure to light; the delirium gives place to coma; "the *powers of speech and deglutition fail;*" the teeth become covered with a foul sordes; the breath offensive; typhoid symptoms, more or less severe, take place, and death closes the scene.

Fourteen deaths occurred in rapid succession, before any *post-mortem*

examination could be obtained. This was a case of a male adult, which terminated on the thirteenth day—of longer duration than any previous case. The symptoms, at times, were so far mitigated as to afford hopes that convalescence would permanently take place; but the flattering and lucid spells proved short and transitory. The same state has been manifested in others. In this case, the vessels of the arachnoid were engorged with dark blood, and the entire membrane covered with a thick purulent matter. Sero-purulent matter was found in the ventricles and at the base of the brain. The medulla oblongata and spinal cord partook of the same lesion, and showed the same traces of diseased action. No lesion was found, either in the lungs, thorax or abdominal viscera. The stomach and bowels appeared healthy, discovering neither inflammation nor ulceration. The results in this case showed most unequivocally the disease to be that denominated, in Europe, cerebro-spinal meningitis. As all the other cases, although not examined autopsically, strictly resembled this and each other, in all their leading features and symptoms, it may, with propriety, be considered to have received its proper appellation.

The case of the boy 5 years of age, Mr. Hall's, which terminated in the short space of ten hours, although the most suddenly fatal, was not taken so violently as either of the others; delirium did not come on until within three or four hours of death. He was sent to town in the morning, and was measured for a garment. During this time the lady noticed he appeared very cold and shivering. A degree of debility was so manifest, that assistance was necessary to extend his arm to finish the measurement. The child, during all this time, made no complaint. Yet the rigors and coldness appeared so great, that the lady was more than half inclined to suspect it might be feigned. He went home himself, when every symptom became more and more aggravated, and death took place at 6 o'clock, on the same evening. All remedies and means used, were utterly unavailing; re-action could not be produced, and never took place; the first collapse proved a fatal one. In this case, the disease run its course in so short a time, that much lesion might not be expected; and though every part was examined scrutinizingly by twelve physicians, it was hard to account for the cause of death, so little diseased action was manifested. As in the other case, great vascularity of the arachnoid was prominent; the substance of the spinal cord, instead of being indurated, as in the other case, was softened almost to a pulp. No purulent matter was found either in the ventricles, or the base of the brain, or spinal canal. The viscera throughout, with the stomach and bowels, were in a perfectly healthy state.

The question naturally arises in this case, where did death commence, what its character and cause? That it is an epidemic, having its cause in the atmosphere, there can be no room to doubt. But why such a deadly epidemic should here arise and hover around this small extent of country, without seeming to spread, there is no means of knowing. There is apparently nothing in the geographical and physical situation of the country, to generate any such cause, any more than in the neighboring towns. *But what can be its cause?* That it is a miasmatic or aerial poison, of a most subtle and deadly nature, every case combines to prove. From the very consideration that every case has been suddenly attacked without any premonitory symptoms, in apparent good health, the striking similarity and uniformity of every symptom, and the onset of the malady, all go to prove this in a most conclusive manner. The characteristic features in every case are such as to show the effect of some sedative and deadly agent upon the cerebro-nervous system. The sudden and extreme collapse, great agony, and indescribable restlessness, that are so uniformily manifested in the congestive fevers of the *miasmatic* districts of the western and southern States, show the cause of the two diseases to be one and the same. But the question will be asked, why is its force of action and lesions confined to the meninges, in this disease, any more than in the general congestive fevers of the malarious districts? This problem we are unable to fathom. We have witnessed death as suddenly in congestive fever of the West, as in the case above mentioned, when no lesions or disorganizations could be found; only an engorgement of the blood vessels of the vital organs, and functional derangement. A poison so sedative in its nature as to paralyze all nervous energy and sensibility to external feeling, is sufficient to suspend the muscles and powers of respiration, arrest the flow of blood to the heart and brain, and produce death by *apnœa*. In this way we account for the death of the boy, whose case is detailed above. That death may be occasioned by the mechanical disarrangement and impediment of the vital machinery, by the sedative action of malaria or other deadly poison, paralyzing the vital energies of the nervous system, is well known to every physician who has practised ten years, either in the southern or western States, however much the idea may be *sneered at here.*

Every case, thus far, has proved fatal, amounting to nineteen or twenty; and no plan of treatment has yet been attended with any success, either in arresting its progress or modifying its paroxysms. We should repose some confidence in liberal doses of the sulphate of quinine, administered immediately on the development of the disease, aided, of course, with strong counter-irritants or the actual cautery itself. All suggestions, however, of this nature, may seem gratuitous at present, as in all probability some further notice will be taken of this epidemic.—*Boston Med. and Surg. Jour*

